# Titanium Dioxide Nanoparticle Penetration into the Skin and Effects on HaCaT Cells

**DOI:** 10.3390/ijerph120809282

**Published:** 2015-08-07

**Authors:** Matteo Crosera, Andrea Prodi, Marcella Mauro, Marco Pelin, Chiara Florio, Francesca Bellomo, Gianpiero Adami, Pietro Apostoli, Giuseppe De Palma, Massimo Bovenzi, Marco Campanini, Francesca Larese Filon

**Affiliations:** 1 Clinical Unit of Occupational Medicine, Department of Medical Sciences, University of Trieste, Via della Pietà 19, Trieste 34129, Italy; E-Mails: mcrosera@units.it (M.C.); andrea.prodi@gmail.com (A.P.); marcella.mauro82@gmail.com (M.M.); bellomo.francesca@gmail.com (F.B.); bovenzi@units.it (M.B.); 2 Department of Chemical and Pharmaceutical Sciences, University of Trieste, Via Giorgeri 1, Trieste 34127, Italy; E-Mail: gadami@units.it; 3 Department of Life Sciences, University of Trieste, Via L. Giorgeri 7/9, Trieste 34127, Italy; E-Mails: mpelin@units.it (M.P.); florioc@units.it (C.F.); 4 Dipartimento di Specialità Medico Chirurgiche, Scienze Radiologiche, Sanità Pubblica, University of Brescia, Piazza del Mercato 15, Brescia 25121, Italy; E-Mails: pietro.apostoli@unibs.it (P.A.); giuseppe.depalma@unibs.it (G.D.P.); 5 IMEM-CNR Institute, Parco Area delle Scienze 37/A, Parma 43124, Italy; E-Mail: marco.campanini@imem.cnr.it

**Keywords:** titanium dioxide, nanoparticles (NPs), *in vitro*, human skin absorption, cytotoxicity

## Abstract

Titanium dioxide nanoparticles (TiO_2_NPs) suspensions (concentration 1.0 g/L) in synthetic sweat solution were applied on Franz cells for 24 h using intact and needle-abraded human skin. Titanium content into skin and receiving phases was determined. Cytotoxicity (MTT, AlamarBlue^®^ and propidium iodide, PI, uptake assays) was evaluated on HaCat keratinocytes after 24 h, 48 h, and seven days of exposure. After 24 h of exposure, no titanium was detectable in receiving solutions for both intact and damaged skin. Titanium was found in the epidermal layer after 24 h of exposure (0.47 ± 0.33 μg/cm^2^) while in the dermal layer, the concentration was below the limit of detection. Damaged skin, in its whole, has shown a similar concentration (0.53 ± 0.26 μg/cm^2^). Cytotoxicity studies on HaCaT cells demonstrated that TiO_2_NPs induced cytotoxic effects only at very high concentrations, reducing cell viability after seven days of exposure with EC_50_s of 8.8 × 10^−4^ M (MTT assay), 3.8 × 10^−5^ M (AlamarBlue^®^ assay), and 7.6 × 10^−4^ M (PI uptake, index of a necrotic cell death). Our study demonstrated that TiO_2_NPs cannot permeate intact and damaged skin and can be found only in the stratum corneum and epidermis. Moreover, the low cytotoxic effect observed on human HaCaT keratinocytes suggests that these nano-compounds have a potential toxic effect at the skin level only after long-term exposure.

## 1. Introduction

Titanium dioxide nanoparticles (TiO_2_NPs) are being widely used in industrial and consumer products due to their strong catalytic activity as compared to their fine-particle counterpart. This characteristic has been attributed to their larger surface area per unit mass, given their smaller size [1].

TiO_2_ is in the top five NPs used in a wide array of consumer products [2], including cosmetics, toothpaste [3], sunscreens [4], and skin treatments for acne vulgaris, condyloma acuminata, atopic dermatitis, hyper-pigmented skin lesions, and other non-dermatologic diseases [5]. TiO_2_ is the most widely used nanomaterial in dermal consumer products [6]. It is also present in paints, foods, and pigments [7]. TiO_2_ appears as a white powder and its nanoparticle formulation is preferred in the cosmetics industry because it avoids white coloration of the skin after application.

Chronic toxicity studies on TiO_2_ focus on the respiratory system, but more effort should be put into studying chronic exposure for topically applied consumer goods, especially with the increase in consumer use of sunscreens that contain TiO_2_NPs [1].

TiO_2_NPs present in cosmetics have the potential to penetrate through the stratum corneum into viable skin layers via intercellular channels, hair follicles, and sweat glands [8]. Several authors have studied the possible penetration of TiO_2_NPs into the skin, using both naked and coated titania samples [9,10,11,12,13], finding that TiO_2_ does not penetrate the skin and the underlying living tissue, remaining on the skin surface or only impregnating the first layers of the stratum corneum. Monterio-Riviere *et al.* [14] showed minimal penetration after UVB exposure *in vitro* and *in vivo* skin. In 2010, Kiss *et al.* [15] investigated the *in vivo* penetration of TiO_2_ on human skin transplanted to immunodeficent mice. They demonstrated *in vivo* that TiO_2_NPs do not penetrate the intact epidermal membrane, but exposed directly to cell culture *in vitro*, they exert significant effects on cell viability. The cytotoxicity of TiO_2_NPs was demonstrated in keratinocytes, using different tests and exposures, with or without UV exposure [1,16,17,18,19], but many *in vivo* experiments on animals did not confirm this effect [1]. More recently, Adachi *et al.* [20] and Wu *et al.* [21] found signs of irritant dermatitis with focal parakeratosis in the stratum corneum and epidermal spongiosis after applying uncoated TiO_2_NPs for long time. Wu *et al.* [21] found that these NPs can penetrate into the deep layer of the viable epidermis in pig ears after 30 days of exposure and into the hairless skin mouse, inducing pathological changes in major organs after 60 days. However, these results were contested by Jonaitis *et al.* [22] who reported methodological deficiencies. Also Sadrieh *et al.* [23] found TiO_2_ in the dermis after 22 days of application of sunscreen creams containing TiO_2_NPs in miniature pigs, but they suspected a contamination.

In most of available study results, after TiO_2_NP dermal exposure, TiO_2_NPs are not significantly systematically available [1]. The lack of penetration through the epidermis is also the main reason for the absence of skin carcinogenesis-promoting effects [24,25]. However, other studies such as Tan *et al.* [26] found, via tape stripping technique, that, levels of TiO_2_NPs in the epidermis and dermis of subjects who applied a sunscreen containing 8% TiO_2_NPs were higher than the levels found in controls,. This difference was not statistically significant given the small sample size. Bannat and Müller-Goymann [27] (applying an oil-in-water emulsion with 5% TiO_2_NPs) found that TiO_2_NPs may be able to penetrate the surface through hair follicles or pores, but no details are given on the fate of such particles.

Adachi *et al.* [20] applied an emulsion containing 10% wt% TiO_2_NPs to the dorsal skin of hairless rats for 56 days and found that the particles were only located in the stratum corneum layer of the epidermis and follicular epithelium. They did not find any evidence of TiO_2_ penetration into viable areas. Moreover, these authors did not find titanium in the internal organ using inductively coupled plasma mass spectroscopy. An increased titanium concentration was found only in the lung samples, probably due to the inhalation of TiO_2_NPs.

It is unlikely that metal oxide nanoparticles penetrate intact human skin under normal conditions, given the tough layer of the stratum corneum, but the impairment of the stratum corneum could increase the skin penetration of nanoparticles [8,28]. Previous *in vitro* data on pig skin treated with tape stripping to remove the stratum corneum [11] did not demonstrate TiO_2_NP skin absorption. However, no data are available on TiO_2_NP absorption using a needle-abraded skin protocol in human skin. It is important to verify if, in a damaged skin condition, TiO_2_NPs fails to cross the skin barrier, as an impairment of the stratum corneum is very common in workers (*i.e.*, wet workers, construction workers, healthcare workers) [29].

To increase the knowledge on this topic, we studied the *in vitro* skin absorption of TiO_2_NPs on intact and damaged human skin with the protocol defined in the European project EDETOX [30] which is used to study other kinds of NPs [31,32,33]. To complete our study, we tested TiO_2_NPs used in skin penetration tests to evaluate their toxicity on short- and long-term exposures (24–48 h and seven days) on keratinocytes.

## 2. Experimental Section

### 2.1. Chemicals

All chemicals were analytical grade. Urea, sodium chloride, sodium hydrogen phosphate, potassium dihydrogenphosphate were purchased from Carlo Erba (Milan, Italy); lactic acid (90% v/v) was bought from Acros Organics (Geel, Belgium); nitric acid (69.5% v/v), hydrogen peroxide (30% v/v), hydrofluoric acid (48% w/v), and ammonium hydroxide (25% w/v) were from Sigma Aldrich (Milan, Italy). Water reagent grade was produced with a Millipore purification pack system (Milli-Q water). The physiological solution used as the receptor phase was prepared by dissolving 2.38 g of Na_2_HPO_4_, 0.19 g of KH_2_PO_4,_ and 9 g of NaCl into 1 L of Milli-Q water (final pH seven.35). The synthetic sweat solution used as donor fluid consisted of 0.5% sodium chloride, 0.1% urea, and 0.1% lactic acid in Milli-Q water; pH 4.5 was adjusted with ammonia.

We used the commercially available TiO_2_ nanopowder (CAS 13463-67-7, n. 677469 provided by Sigma Aldrich—Milan, Italy).

### 2.2. Nanoparticle Characterization

The TiO_2_ NPs were investigated by Transmission Electron Microscopy (TEM) once they were dispersed in synthetic sweat and at the end of the experiments (after the 24 h exposure time) to visualize the dimensions of the NPs and the aggregation state of the donor phase. In addition, by performing electron diffraction experiments the crystalline phases of the TiO_2_ nanopowder were checked. In agreement with the Raman characterization reported in [34], the dominant phase observed for the NPs is anatase (>90%), while a small amount of the NPs show a rutile structure.

Since the behavior and the aggregation state of the NPs in different mediums depend strongly on the surface charge of the NPs and the ionic strength of the suspension, a further characterization using both Dynamic Light Scattering and Z-potential techniques has been carried out. The investigation was performed using the 90Plus PALS instrument (Brookhaven Corp., Holtsville, NY, USA). In Alinovi *et al.* 2015 [34] the complete characterization is reported.

Finally, in order to evaluate the ions released from the NPs once they were put in synthetic sweat, 4 mL of the donor phase was ultra-filtered using the Amicon Ultra-4 centrifugal filters (10K MWCO). The ultrafiltration has been performed in centrifuge at 5000 rpm for 30 min in order to remove the NPs, but not eventual titanium ions, from the solution. The solution has been analyzed by ICP-AES to quantify the titanium concentration. The ultrafiltration has been repeated on three different aliquots at the beginning of the permeation experiments and on three other aliquots at the end of the 24-h exposure time. The titanium concentration was always below the limit of detection.

### 2.3. Preparation of Skin Membranes

Human abdominal full thickness skin was obtained as surgical waste from patients aged 45–65 years. After the skin excision, subcutaneous fat was removed with a scalpel blade and hair shaved from the epidermal layer, then skin samples were stored at −25 °C for a period up to, but not exceeding, 4 months. It has been demonstrated that this procedure does not damage skin barrier properties. At the day of the experiment, skin samples were defrosted in physiological solution at room temperature for a 30 min period and then 4 × 4 cm^2^ pieces were cut from each skin specimen and mounted separately on the diffusion cells. Damaged skin samples were obtained using a needle-abrasion technique described elsewhere [35]. Skin integrity was tested before and after each experiment using electrical conductibility by means of a conductometer (Metrohm, 660, Metrohm AG Oberdorfstr. 68 CH-9100 Herisau) operating at 300 Hz and connected to two stainless steel electrodes [36]. The conductibility data in µS were converted into KΩ/cm^2^. Cells with a resistance lower than 3.95 ± 0.27 KΩ/cm^2^ were considered to be damaged and rejected as suggested by Davies *et al.* [37].

### 2.4. In Vitro Diffusion System

Percutaneous absorption studies were performed using static diffusion cells following the Franz method [38]. The receptor compartment had a mean volume of 14.0 mL and was maintained at 32 °C by means of circulation of thermostated water in the jacket surrounding the cell. This temperature value was chosen in order to reproduce the hand physiological temperature at normal conditions. The physiological solution used as the receptor phase was prepared by dissolving 2.38 g of Na_2_HPO_4_, 0.19 g of KH_2_PO_4_, and 9 g of NaCl into 1 L of Milli-Q water (final pH 7.35).

The concentration of the salt in the receptor fluid was approximately the same that can be found in the blood. The physiological solution used as the receiving phase was continuously stirred using a Teflon-coated magnetic stirrer. Each piece of skin was clamped between the donor and the receptor compartment; the mean exposed skin area was 3.29 cm^2^ and the average membrane thickness was 1 mm. Two different experiments were conducted using intact (experiment 1) and damaged skin (experiment 2) as described below.

### 2.5. Experiment 1

The donor phase was prepared just before the experiment using a sonicated suspension of TiO_2_NPs at a concentration of 1.0 g/L dispersed in synthetic sweat at pH 4.5 to reproduce the *in vivo* condition. The TiO_2_ concentration in the donor phase was confirmed by Inductively Coupled Plasma-Atomic Emission Spectroscopy (ICP-AES) analysis prior to the test.

At time 0, the exposure chambers of 6 Franz diffusion cells were mounted with intact skin samples and filled with 2.5 mL of the donor suspension (606 μg/cm^2^) to ensure an infinite dose. The experiment was run for 24 h, and during this period, 1.5 mL of the dermal bathing solution was removed at selected intervals (4, 8, 16, 24 h) and analyzed. Each receptor sample was immediately replaced with an equal volume of fresh physiological solution. At 24 h the dermal bathing solution and the donor phase of each diffusion cell were recovered for the following analysis.

### 2.6. Experiment 2

Experiment 1 was repeated using an abraded skin protocol as suggested by Bronaugh and Steward [35]. Skin was abraded by drawing the tip of a 19-gauge hypodermic needle across the surface (20 marks in one direction and 20 perpendicular).

### 2.7. Blanks

For each experiment, two cells were added as blanks. The blank cells were treated as the other cells with the exception that only synthetic sweat was used in the donor compartment.

### 2.8 Skin Digestion after the Experiment

After the experiment, the skin pieces were washed three times with physiological solution to remove TiO_2_NPs on the skin, then removed from the diffusion cells and treated as follows: Skin samples from exp. 1 were separated into epidermis and dermis by heat shock immerging in water at 60 °C for 1 min before freezing, while skin samples from exp. 2 were simply stored in a freezer at −25 °C. At the time of the analysis, the skin membranes were dried for 2 h at room temperature, then cut into sections, weighed, and put into 100 mL disposable Digitubes^TM^ with 10 mL of HNO_3_ and 2 mL of H_2_O_2_ for digestion. They were heated for 24 h at 90 °C in a block heater (SPB 100-12, PerkinElmer), then 0.2 mL of HF was added, and heated until the remaining solutions were of 2 mL in volume. The solutions were diluted to a volume of 10 mL with Milli-Q water for the analysis with ICP-AES.

### 2.9. Analytical Measurements

The metal concentrations in the receiving phase skin were determined by Zeeman corrected graphite furnace atomic absorption spectrophotometry (GF-AAS) using a Varian Duo instrument (GTA 120, AA 240 Z). The calibration standards were prepared by standard solutions of single elements ranging from 0.5 to 1000 µg/L: Titanium in H_2_O atomic absorption standard solution (Sigma-Aldrich, Milwaukee, WI, USA). The limit of detection (LOD) calculated as three standard deviations of the background signal obtained on 10 blind samples at the operative wavelength of 364.3 nm was 5 µg/L. The precision of the measurements as relative standard deviation (RSD%) for the analysis was always less than 5%.

Total titanium concentration in the donor phases and in the solutions resulting from the mineralization of the skin samples were performed by Inductively Coupled Plasma-Atomic Emission Spectrometry (ICP-AES) using a Spectroflame Modula E optical plasma interface (OPI) instrument (by SPECTRO, Germany). The analyses were conducted using a calibration curve obtained by dilution (range: 0–10 mg/L) of titanium standard solution for ICP-AES analyses (by Teknolab A/S, Norway). The limit of detection (LOD) at the operative wavelength of 334.941 nm was 20 µg/L. The precision of the measurements as relative standard deviation (RSD%) for the analysis was always less than 5%.

### 2.10. Cell Tests

#### 2.10.1. Cell Culture

The immortalized human keratinocyte HaCaT [39] cell line was purchased from Cell Line Service (DKFZ, Eppelheim, Germany). Cells were cultured in high-glucose Dulbecco’s Modified Eagle’s medium (DMEM) supplemented with 2 mM L-Glutamine, 100 U/mL penicillin-100 µg/mL streptomycin, and 10% fetal bovine serum (FBS) at 37 °C in a 5% CO_2_ atmosphere. Cells received fresh medium every 3 days and were subcultured every seven days.

Stock solutions of TiO_2_NPs (1000 µg/mL ethanol) were diluted to the required concentrations (1.5 × 10^−7^–1.0 × 10^−3^ M equal to 0.007–50 μg/cm^2^) in the cell culture medium and sonicated before use.

#### 2.10.2. MTT Assay

Cells (5 × 10^3^ cells/well) were plated in 96-well plates for 24 h and then exposed to TiO_2_NPs (1.5 × 10^−7^–1.0 × 10^−3^ M equal to 0.007–50 μg/cm^2^). After 24, 48 h, and seven days of exposure, a 10% MTT solution was added and, after 4 h, the insoluble crystals were solubilized with DMSO [40]. Plates were read in a Microplate Autoreader (Bio-Tek Instruments) at 540/630 nm. Data are reported as percentages of control and are the mean ± SE of four independent experiments performed in triplicate.

Unspecific reactions of TiO_2_NPs with MTT reagents was excluded by preliminary experiments.

Furthermore, two washes were performed after TiO_2_ cell treatments and before MTT. This procedure should in any case avoid or drastically reduce false positive results deriving from unspecific reactions between TiO_2_ and the dyes.

#### 2.10.3. AlamarBlue^®^ Assay

Cells (15 × 10^3^ cells/well) were cultured in 96-well plates and, after 24h, exposed to TiO_2_NPs (1.5 × 10^7^–1.0 × 10^−3^ M equal to 0.007–50 μg/cm^2^) for 24, 48 h, and seven days. After 4 h of incubation with the AlamarBlue^®^ reagent, fluorescence intensity was read by a FluoroCount Microplate Fluorometer (Packard, Germany) at an excitation wavelength of 530 nm and emission wavelength of 590 nm. Data are reported as a percentage of control and are the mean ± SE of four independent experiments performed in triplicate.

Unspecific reactions of TiO_2_NPs with AlamarBlue^®^ reagents were excluded by preliminary experiments.

Furthermore, two washes were performed after TiO_2_ cell treatments and before AlamarBlue^®^. This procedure should in any case avoid or drastically reduce false positive results deriving from unspecific reactions between TiO_2_ and the dyes.

#### 2.10.4. Propidium Iodide Uptake

Cells (1 × 10^5^ cells/well) were seeded in 96-well plates and, after 24 h, exposed to TiO_2_NPs (1.5 × 10^−7^–1.0 × 10^−3^ M equal to 0.007–50 μg/cm^2^) for seven days. Propidium iodide (PI) uptake was performed as previously described [41,42]. Briefly, after treatment, cells were rinsed with 200 μL of 3.0 × 10^−6^ M PI in phosphate-buffered saline (PBS) and fluorescence intensity was read by a FluoroCount Microplate Fluorometer (Packard, Germany) at an excitation length of 530 nm and emission length of 590 nm after 30 min.

All samples were subsequently permeabilized with 1% Triton-X for 30 min to obtain total cell content for each sample and fluorescence read. Positive control was obtained by permeabilizing untreated cells with 1% Triton-X. Data are reported as % of positive control (equal to 100% PI uptake) after normalization on total cell content and are the mean ± SE of three independent experiments performed in triplicate.

### 2.11. Statistical Analysis

Ti concentration data (μg/cm^3^) in the receptor solution were converted to the total amount that penetrated (μg/cm^2^), with a correction for dilution due to sample removal.

Data analysis were performed with Excel 2007 for Windows, and Stata Software, version 11.0 (StataCorp LP, College Station, TX, USA). Skin absorption data were reported as mean ± standard deviation (SD). The difference among independent data was assessed by means of the Mann-Whitney test.

Cytotoxicity data were reported as mean ± standard error (SE) of at least three independent experiments performed in triplicate. The concentration giving 50% of the maximal effect (EC_50_) was calculated using GraphPad software version 4.0 (Prism GraphPad, Inc.; San Diego, CA, USA). A *p* < 0.05 was considered as significant.

## 3. Results and Discussion

### 3.1. Nanoparticles Characterization

**Figure 1:**
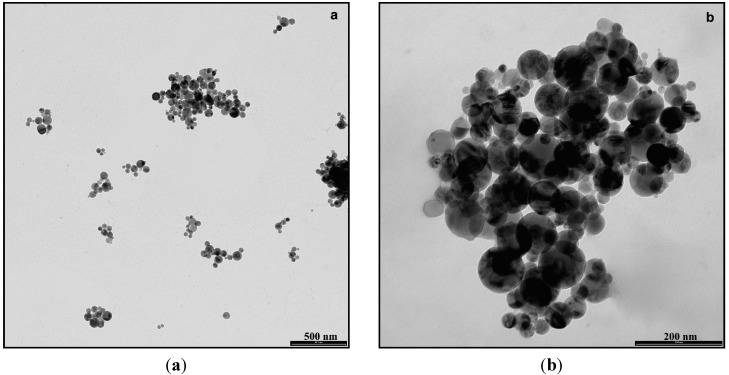
Representative Transmission Electron Microscopy images of agglomerated TiO_2_NPs dispersed in synthetic sweat at the beginning of the experiments (Bar: (**a**) = 500 nm, (**b**) = 200 nm).

The characterization performed on TiO_2_NP specimens showed that the NP_S_ have a regular spherical shape and appear as slightly aggregated (Figure 1).

**Figure 2:**
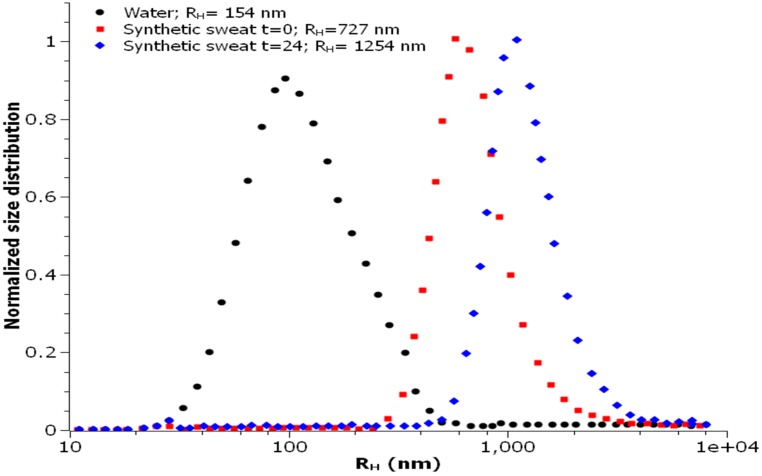
Size distribution of TiO_2_NPs in water and synthetic sweat suspension, estimated by DLS (Dynamic Light Scattering).

The size distribution is centered on the value of 38 nm. The hydrodynamic radius value (RH) observed in water was centered on 154 nm, while it increased considerably when assessed in synthetic sweat, reaching a value of 727 nm (Figure 2) at time 0 and 1254 nm after 24 h.

The observed behaviors were in agreement with the measured Z-potential values, reported in Table 1. The measured surface charge values suggest that the NPs dispersed in water are stable thanks to an electrostatic stabilization; for this medium, the small R_H_ value highlighted the presence of small aggregates (R_H_ ≈ 150 nm) constituted by few NPs.

**Table 1:** Comparison of Z-potential values in water and in synthetic sweat (mean ± SD).

Medium Specimen	Water	Synthetic Sweat t = 0	Synthetic Sweat t = 24 h
**TiO_2_**	−31.7 ± 1.02 mV	−36.8 ± 3.8 mV	−19.0 ± 4.1 mV

When the nanoparticles were dispersed in synthetic sweat, on the contrary, they immediately started to agglomerate in bigger clusters (R_H_ ≈ 730 nm), as demonstrated by DLS measurements, even if it was not detected by any immediate change in the surface charge.

A remarkable change in the surface charge, due to the absorption of ionic species on the NPs surface, was, however, observed after 24 h. In particular, the significant reduction of the Z-potential was effective in hindering the electrostatic stabilization, thus leading to a strong increase in the mean hydrodynamic radius as detected by DLS measurements (R_H_ ≈ 1260 nm).

### 3.2. Franz Diffusion Cells Experiments

After 24 h of exposure, the average concentration of Ti in the receiving solution was below the level of detection (LOD) of 5 μg/L for both intact and damaged skin.

**Figure 3:**
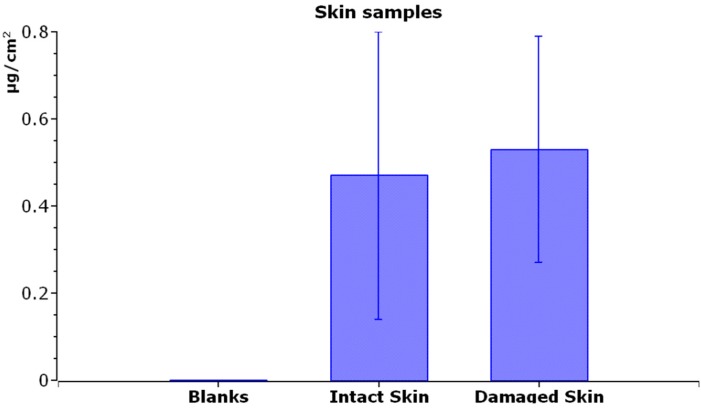
Titanium content (µg/cm^2^) inside the skin (epidermis + derma) of blank cells (exposed to physiological solution), intact skin, and damaged skin (exposed to TiO_2_NPs). Mean and standard deviation of six cells each.

As shown in Figure 3, the average amount of Ti in intact skin, after 24 h of exposure, was 0.47 ± 0.33 μg/cm^2^ in the epidermal layer, while the dermal layer concentration was below LOD.

Damaged skin, evaluated as a whole, has shown a similar concentration (0.53 ± 0.26 μg/cm^2^) to the Ti content of intact skin (Figure 3).

### 3.3. Effect of TiO_2_NPs on Cell Viability

**Figure 4:**
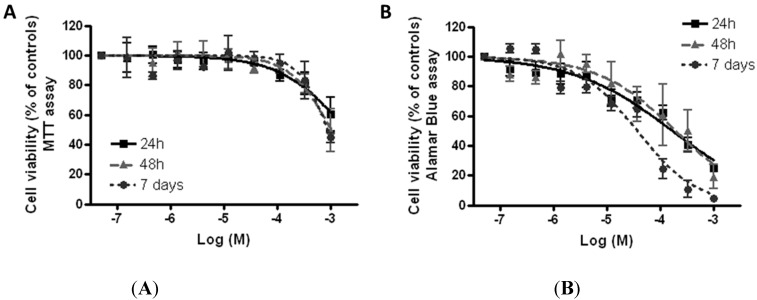
Cytotoxicity of TiO_2_NPs. Cell viability was measured by MTT assay (**A**) and AlamarBlue^®^ assay (**B**) after 24 h, 48 h, and seven days of exposure to TiO_2_NPs (1.5 × 10^−7^–1.0 × 10^−3^ M equal to 0.007–50 μg/cm^2^) on HaCaT cells. Data are reported as % of untreated controls (equal to 100% cell viability) and are the mean ± SE of four independent experiments performed in triplicate.

The cytotoxic effect induced by TiO_2_NPs on HaCaT skin keratinocytes was evaluated using the MTT reduction assay and the AlamarBlue^®^ assay. Figure 4A shows the concentration-response curves obtained after 24, 48 h, and seven days of exposure to TiO_2_NPs (1.5 × 10^−7^–1.0 × 10^−3^ M equal to 0.007–50 μg/cm^2^) obtained by the MTT assay. TiO_2_NPs induced a very low cytotoxic effect that was significant (*p* < 0.05) for concentrations higher than 1.1 × 10^−4^ M for all the exposure times considered. Intriguingly, cytotoxicity was independent of the length of exposure, since the effects observed after 24, 48 h, or seven days were almost overlapping. After seven of days of exposure, TiO_2_NPs reduced cell viability with an EC_50_ equal to 8.8 × 10^−4^ M (95% confidence limits, CL = 6.2–12.4 × 10^−4^ M) corresponding to 44 μg/cm^2^ (95% confidence limits, CL 31–62 μg/cm^2^).

Figure 4B shows the effect of TiO_2_NPs on cell viability evaluated by the AlamarBlue^®^ assay. The cytotoxic effect was slightly higher with respect to that evaluated by the MTT assay. In particular, the TiO_2_NP-induced cytotoxic effect was significant (*p* < 0.05) at concentrations higher than 1.2 × 10^−5^ M for all the exposure times considered. The highest effect was achieved after seven days of exposure, after which TiO_2_NPs reduced cell viability with an EC_50_ of 3.8 × 10^−5^ M (95% CL = 2.6–5.3 × 10^−5^ M), equal to 1.9 μg/cm^2^ (95% CL = 1.3–2.7 μg/cm^2^).

### 3.4. Effect of TiO_2_NPs on Membrane Damage

**Figure 5:**
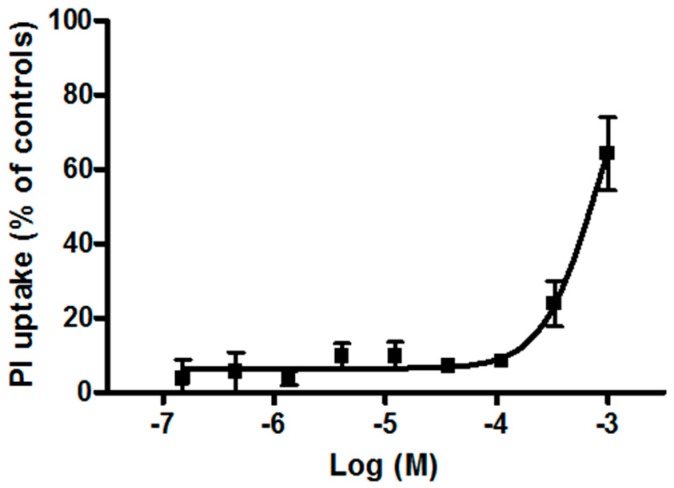
PI uptake in HaCaT cells exposed for seven days to TiO_2_NPs (1.5 × 10^−7^–1.0 × 10^−3^ M). Data are reported as mean ± SE of three independent experiments performed in triplicate.

The plasma membrane damage induced by TiO_2_NPs was evaluated after seven days of NP exposure by propidium iodide (PI) uptake. As shown in Figure 5, TiO_2_NPs induced a concentration-dependent PI uptake starting from 1.1 × 10^−4^ M, with an EC_50_ value of 7.6 × 10^−4^ M (95% CL = 6.2–9.4 × 10^−4^ M) equal to 38 μg/cm^2^ (95% CL = 31–47 μg/cm^2^).

### 3.5. Discussion

No titanium permeation was demonstrated after 24 h of exposure of the skin to TiO_2_NPs both in intact and in damaged skin. In the skin, titanium was detectable only in the epidermis. Since the total amount of NPs was similar in both intact and damaged skin, we can assume that lesions do not increase permeation (at least with current limits of detection).

Our study is in accordance with other authors [9,10,11,12,13] who denied the TiO_2_ capability to permeate the human skin. This is due to the great stability of the TiO_2_NPs and its negligible ionization in the physiological condition, which leads to an accumulation of NPs on the surface of the skin. Moreover, the big size of the particles and their tendency to form aggregates further reduce the skin absorption capability [8].

To evaluate the toxic potential of TiO_2_NPs at the skin level, a preliminary study was carried out on human HaCaT keratinocytes, an accepted *in vitro* model for the screening of the cutaneous toxicity of compounds [43]. On HaCaT cells, TiO_2_NPs induced a slight cytotoxic effect, reducing cell viability with EC_50_ values equal to 8.8 × 10^−4^ M (equal to 44 μg/cm^2^, MTT assay) and 3.8 × 10^−5^ M (equal to 41.9 μg/cm^2^, AlamarBlue^®^ assay) after seven days of exposure. These results are in line with previous studies carried out on HaCaT cells reporting the ability of TiO_2_NPs to induce oxidative stress and mitochondrial damage [16,19,44]. However, our results suggest a low toxic potential of TiO_2_NPs since the effects were significant starting from the concentration of 1.1 × 10^−4^ M (MTT assay).

Intriguingly, no dependency on the exposure time was observed in the MTT test, which, on the contrary, was appreciable in the AlamarBlue^®^ assay. This discrepancy could be tentatively ascribed to the relatively higher sensitivity of the AlamarBlue^®^ assay with respect to the MTT test [45]. After seven days of contact, TiO_2_NPs also induced a significant increase of PI uptake, suggesting a cytotoxic effect related to membrane damage and compatible with necrosis or late-apoptotic cell death, as recently reported [44]. Notably, this effect occurred with an EC_50_ value (7.6 × 10^−4^ M, equal to 38 µg/cm^2^) very close to that obtained in the MTT assay after long-term exposure (*i.e.*, seven days). One speculative explanation could account for the low cytotoxic potential of TiO_2_NPs that can be appreciable only at very high concentrations and possibly due to induction of oxidative stress followed by cell death. On the whole, the present results are in line with previous studies reporting low toxicity (see references above), as evaluated by cell viability and morphology after short-term exposure to TiO_2_NPs [46], and lack of phototoxicity, acute cutaneous irritation, or skin sensitization [47], and the results strengthen the notion of the low risk associated to these NPs.

Our study demonstrated that TiO_2_NPs cannot permeate intact and damaged skin and can exert a low cytotoxicity effect only at a high dose and long exposure. Nevertheless, our study has some limitations. The first limitation is the *in vitro* design of our study that can verify only passive diffusion through the skin, while active penetration could happen in the *in vivo* condition. The second is the short-term run of experiments (24 h) while in real conditions workers and consumers are exposed to TiO_2_NP-containing products for months and years. The third limitation is the use of HaCaT cells instead of normal human epidermal keratinocytes. We chose these cells because they are easy to culture and because our results can be compared with many similar experiments done on testing nanoparticles on these cells. Nevertheless, they are karyotypically/genetically unstable and additional experiments must be done using human epidermal keratinocytes.

Further studies for the safety evaluation of TiO_2_NPs in sunscreens are needed, simulating real-world exposure conditions (sunburned skin and UV exposure) on users to verify if a long-term exposure can cause local or systemic effects [48].

## 4. Conclusions

We did not find permeation of TiO_2_NPs in either intact or damaged skin. We located NPs in the epidermal layer but not in the dermal layer, and the skin concentration was similar in both tests: skin lesions do not appear to alter the permeation of these NPs.

These results can be explained by the great stability and low ionizing capacity of these particles and are in accordance with several studies in the literature. On the whole, the absence of TiO_2_NP permeation both in intact and in damaged skin, as well as the low cytotoxicity observed on human HaCaT keratinocytes, suggested a low toxic potential of these nano-compounds at the skin level. Moreover, further studies for the safety evaluation of TiO_2_NPs in sunscreens are needed, simulating real-world scenarios on sunburned skin and with UV exposure in long-term chronic exposure conditions.
